# More Lack in the World: The Complex Connection between Undernutrition and Climate Change

**DOI:** 10.1289/ehp.119-a524a

**Published:** 2011-12-01

**Authors:** Angela Spivey

**Affiliations:** Angela Spivey writes from North Carolina about medicine, environmental health, and personal finance.

Anthropogenic climate change is projected to reduce cereal yields and food security and therefore to undermine future efforts to reduce child undernutrition. But models are needed to better measure the potential impacts of climate change on population health. Now researchers have developed a model to estimate future undernutrition attributable to climate change as a function of its impact on crop productivity [*EHP* 119(12):1817–1823; Lloyd et al.].

Undernutrition is measured using criteria such as stunting (smaller-than-average height-for-age) and underweight (smaller-than-average weight-for-age). The researchers developed and validated the model using previously published data about past food availability, the prevalence of stunting, and gross domestic product. Then they used projections of future calorie availability under two climate change scenarios and a reference scenario of no climate change to estimate undernutrition among children under age 5 years in five regions of South Asia and sub-Saharan Africa in 2050.

The model estimates that climate change will lead to an average relative increase in moderate stunting (height that is more than two standard deviations below the expected height-for-age) of 1–29%, depending on region, compared with a future without climate change. Climate change will have a greater impact on rates of severe stunting (height more than three standard deviations below the expected mean), which the authors estimate will increase by an average of 31–62%, depending on region.

**Figure:**
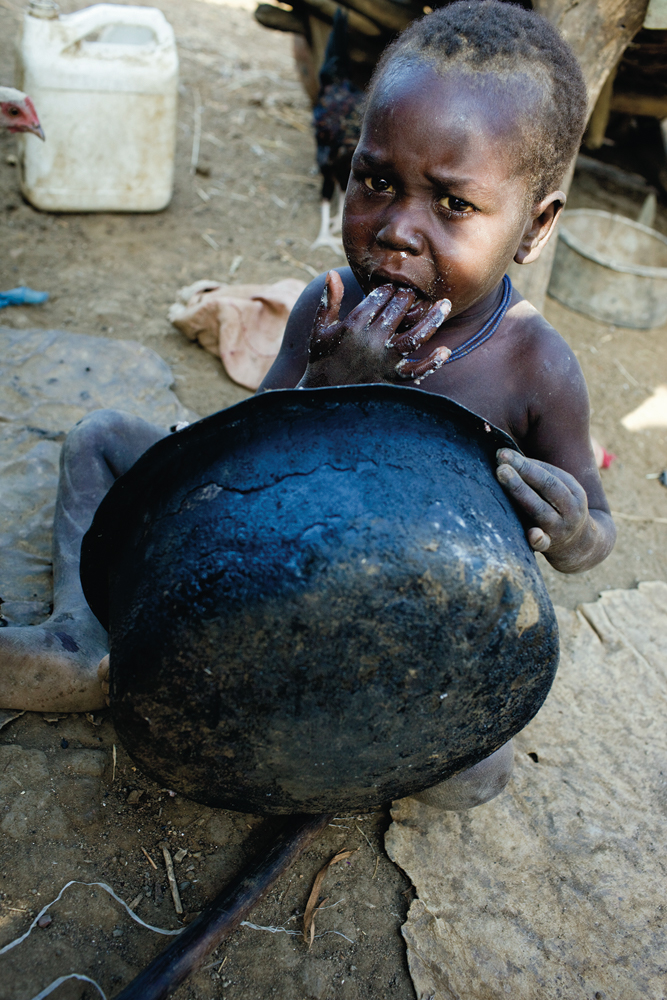
A Ugandan child scrapes out extra food from a cooking pot. Climate change is expected to undermine efforts against child undernutrition by reducing cereal yields and food security. © Mikkel Ostergaard/Panos Pictures

Climate change is likely to affect undernutrition through a variety of means in addition to crop production, including impacts on infectious diseases in humans, plant pests and diseases, labor productivity, and water availability. One limitation of the current study is the difficulty in quantifying the impact of climate change in the face of uncertainty about how countries will develop and manage their food systems. The authors state that their current study illustrates the importance of the outcome used to predict impacts—undernourishment (lack of food) versus stunting, for instance, or moderate versus severe stunting have different implications for decision making and for population health.

The study adds to the evidence suggesting that climate change is likely to increase future hunger and undernutrition even under optimistic assumptions of future emissions and economic growth. The study results suggest that to reduce and prevent future undernutrition, it is necessary to not only reduce emissions of greenhouse gases but also increase food access and improve socioeconomic conditions.

